# Association between dental age and malocclusions: a systematic review

**DOI:** 10.1186/s12903-024-04143-7

**Published:** 2024-03-25

**Authors:** Gabriela Fonseca-Souza, Amanda Renostro-Souza, Lhorrany Alves-Souza, Geraldo Thedei Junior, Maria Angélica Hueb de Menezes-Oliveira, Lívia Azeredo Alves Antunes, Svenja Beisel-Memmert, Christian Kirschneck, Juliana Feltrin-Souza, Erika Calvano Küchler

**Affiliations:** 1 Department of Stomatology, Federal University of Paraná, Av. Prefeito Lothário Meissner 632, Jardim Botânico, Curitiba, Paraná 80210-170 Brazil; 2 Department of Biomaterials, University of Uberaba, Av. Nenê Sabino 1801, Bairro Universitário, Uberaba, Minas Gerais 38055-500 Brazil; 3 Department of Specific Formation, Fluminense Federal University, Rua Dr. Silvio Henrique Braune 22, Centro, Nova Friburgo, Rio de Janeiro, 28625‑650 Brazil; 4 Department of Orthodontics, Medical Faculty, University Hospital Bonn, Welschnonnenstr. 17, Bonn, 53111 Germany

**Keywords:** Malocclusion, Age determination by teeth, Orthodontics, Dental development, Dental age, Systematic review

## Abstract

**Background:**

The evidence in the literature suggests that some skeletal or dental malocclusions are involved with dental development, resulting in advanced or delayed dental age (DA). The purpose of this systematic review was to investigate the association between DA and different types of malocclusions.

**Methods:**

The search was carried out on PubMed, Scopus, Web of Science, Virtual Health Library, and in the gray literature. Observational studies that evaluated the association between DA and sagittal, vertical, or transversal malocclusions were included. The quality assessment was performed using the Newcastle–Ottawa Scale (NOS). The data from primary studies were narratively synthesized. The certainty of evidence was evaluated using the GRADE approach. The study was conducted from August 2023 to October 2023.

**Results:**

One Thousand Nine Hundred Ninety-One records were identified in the initial search. Twenty (*n* = 20) studies were included. Most of the studies (*n*=15) presented a moderate quality according to NOS. Twelve studies evaluated the association between DA and sagittal discrepancies; eight studies evaluated vertical discrepancies, and only one study analyzed a transversal discrepancy. Demirjian’s method for DA assessment was the most used among the studies. The primary studies observed that patients of both sexes presenting a vertical growth pattern and males with skeletal Class III malocclusion tend to have advanced DA. The study that investigated transversal malocclusion found that unilateral posterior cross-bite is associated with delayed DA. The certainty of evidence was very low for all outcomes evaluated.

**Conclusion:**

DA may be associated with the type of malocclusion. It is suggested that DA can be used as an initial diagnostic tool in orthodontics. Future well-designed studies should be performed in order to investigate the association between DA and different types of malocclusions in more detail.

**Trial registration:**

This study was registered in the PROSPERO database (CRD42023454207).

**Supplementary Information:**

The online version contains supplementary material available at 10.1186/s12903-024-04143-7.

## Background

Dental age (DA) is a biological age marker that plays an important role in many fields, including forensic science, and clinical practice, such as in the pediatric dentistry and orthodontics [1]. In the forensic field, DA is mainly used in cases of reconstructive identification [2]. In the daily clinical practice, data about a patient’s maturation influence the diagnosis and treatment plan in orthodontics and pediatric dentistry [3]. Individuals with the same chronological age (CA) can present variations in the developmental stages of different systems. Thus, the estimation of biological age markers such as skeletal maturation and DA may better clinically describe the developmental status of a patient [4]. The evaluation of DA is performed by measuring the degree of eruption or developmental stage of teeth [5, 6]. The analysis of developmental stages is considered more reliable for DA estimation than tooth eruption as this process is susceptible for disruptions by several factors, such as ankylosis, supernumerary teeth, delayed exfoliation of the primary teeth, and impaction [7]. There are several different methods to determine DA, including Demirjian, Willems, Cameriere, and Nolla. Demirjian is the most widely used [8].

Malocclusions are a set of human craniofacial morphologic characteristics that may vary from minor to major alterations of dental or skeletal origin. They are divided into three groups: sagittal, vertical, and transverse discrepancies [9]. Sagittal patterns include class I, II and III malocclusions [10]. Vertical discrepancies are related to an increased or reduced vertical dimension of the face, including open and deep bites [11]. The transverse discrepancy is associated with dental arch width and includes crossbite [9]. Clinically, in orthodontic practice, the type of malocclusion determines the treatment planning decisions.

There is some evidence in the literature that DA and skeletal malocclusion may be biologically related [12]. The formation of the jaws and teeth are intimately related due to their common embryological origin, shared regulatory mechanisms and genetic factors [12]. Some studies suggested that some skeletal or dental malocclusions are involved with the dental development, resulting in advanced or delayed DA [13–16]. However, the results presented are not consistent. Therefore, the aim of this systematic review was to evaluate the association between DA and different types of malocclusions.

## Methods

### Protocol, registration and research question

This systematic review was registered in the International Prospective Register of Systematic Reviews (PROSPERO)[17] (registry number: CRD42023454207) and reported following the Preferred Reporting Items for Systematic Review and Meta-Analysis (PRISMA) [18]. The study was conducted from August 2023 to October 2023.

The research question was: Does DA differ in different types of malocclusions (sagittal, vertical, and transversal discrepancies)?

### Search strategy

The articles were searched electronically in PubMed, Scopus, Web of Science, Embase, and Virtual Health Library. A search was also performed using sources of gray literature, such as CAPES thesis databases, Open Gray, and abstracts from the International Association of Dental Research (IADR). The references list of the primary studies that matched the inclusion criteria were also assessed. No language or time of publication restrictions were established.

The search strategy was based in terms related to malocclusion and DA. For the exposure, the Medical Subject Heading (MeSH Terms) were "Dental Occlusion", "Malocclusion", "Dental Arch", "Malocclusion, Angle Class I", "Malocclusion, Angle class II", and "Malocclusion, Angle Class III"; and the free keywords were "Orthodontic treatment, "Orthodontics, "Skeletal Malocclusion, "Occlusal alteration”. The MeSH terms related to the outcome included "Tooth Calcification", "Age Determination by Teeth", and "Odontogenesis"; and the free keywords were "Dental age", "Dental maturation", "Dental development", "Demirjian", "Nolla", and “Willems". The set of terms for each concept was combined using the Boolean operator “OR” and the concepts were combined with the Boolean Operator “AND” (see Additional file 1).

### Eligibility criteria

Inclusion criteria were observational studies (cross-sectional, case-control, and cohort) that evaluated the association between DA and malocclusions (sagittal, vertical, and transverse). However, no case-control or cohort studies met the eligibility criteria; thus, only cross-sectional studies were included in this systematic review. Exclusion criteria were clinical trials, editorial letters, pilot studies, literature reports, *in vitro* studies, animal experiments, and case of series. Studies that included individuals with syndromes or craniofacial anomalies were also excluded.

### Study selection and data collection

The references identified through the search strategy were exported into EndNote X9® (Clarivate Analytics, USA). Duplicate studies were identified and excluded. Then, 3 trained and independent reviewers selected the studies by title and abstract. Any disagreement was solved by consensus among the reviewers and consulting an experienced fourth reviewer. Then, the full-text articles were analyzed, and the relevant information was extracted through a data extraction form containing information on author, year of publication, country, study design, participants’ mean age, total number of participants, percentage of male participants, local of recruitment, methods to obtain data, criteria for DA evaluation, exclusion criteria and main results. When the primary studies did not report enough data or missing data, it was tried to contact the authors. In the absence of response for the requested data, the study was excluded, or the missing results were described as “not reported” (NR).

### Quality assessment

The quality assessment was performed using the Newcastle−Ottawa Scale (NOS) [19]. An adapted version of NOS was used for cross-sectional studies [20]. This version presents three dimensions with seven items and is based on a star system as follows: selection (4 items and maximum 5 stars), comparability (1 item and maximum 2 stars), and outcome (2 items and maximum 3 stars) [20]. In the selection dimension, the size and representativeness of the sample, comparability between respondents and non-respondents, and the description of the criteria used to determine the exposition (malocclusion) were considered; in the comparability dimension, the presence of controls for the most important factor and for additional factors was evaluated; and in the outcome dimension, we considered whether the examiners were trained to determine the outcome (DA) and whether they were blinded in relation to the type of malocclusion of the patient. Besides that, the description and applicability of the statistical tests used was taken into account. Then, the studies awarded with 0 to 4, 5 to 6, and > 7 stars were classified as having low, moderate, and high quality, respectively. Two independent reviewers performed this step, and any disagreement was resolved by consensus.

### Summary measures and data-synthesis

To analyze the association between DA and malocclusions, the types of malocclusions were categorized into sagittal, vertical, and transversal discrepancies. The data from primary studies were narratively synthesized considering the type of malocclusion evaluated (sagittal, vertical, or transversal; skeletal or dental malocclusion), the classification used to determine the type of malocclusion, the method used to evaluate DA, the sample’s mean CA and DA, the difference between DA and CA, the standard deviations, and the description of the main results of each study. Furthermore, when available, the data were synthesized according to the patient’s gender.

It was observed that the primary studies used different terms to classify vertical discrepancies. Some used “vertical growth pattern” and “horizontal growth pattern”, while others used “long face” and “short face”. To standardize nomenclatures, we used the terms vertical and horizontal growth patterns.

Considering that the primary studies adopted different criteria to evaluate DA, evaluated different malocclusions or used different methods to classify the malocclusion, and data regarding the DA was incompletely presented in several studies, a meta-analysis was not possible.

### Certainty of evidence

The certainty of the evidence for each outcome was evaluated using the Grading Recommendations Assessment, Development and Evaluation (GRADE) approach [21, 22], through the online tool GRADEpro/GDT (https://gdt.gradepro.org/ app) [23]. For observational studies, the GRADE approach has five domains that can decrease the certainty of bias (risk of bias, inconsistency of results, indirectness of evidence, imprecision of results, and publication bias) and three domains that can increase the certainty of evidence (large effects, dose–response gradient, and plausible confounding effect). Usually, in this approach, the results estimated by a meta-analysis are used to rate the domains [22]. However, in the present study, the evidence was only summarized narratively, so the criteria proposed by Murad et al. [24] for systematic reviews with no meta-analysis were used to rate the GRADE’s domains as follows: risk of bias rating was based on the methodological quality of the primary studies (low, moderate or high); inconsistency was evaluated according to the direction the effect varied across the primary studies (similar or contrasting results); indirectness was rated according to the direct evidence provided by the primary studies for the research question; for imprecision, we considered the number of patients included in all studies (optimal information size – OIS), which should be of at least 400 individuals; publication bias was suspected when the body of evidence consisted of only small positive studies or when studies are reported in trial registries but not published; large effect, plausible confounding and dose-response gradient were not rated since none of them were noted in the primary studies included.

Based on the rating of the GRADE’s domains, the certainty of the evidence was graded into four levels (high, moderate, low, and very low), which reflect the confidence that the estimated effect is close to the true effect [22].

## Results

### Study selection

**Fig. 1:**
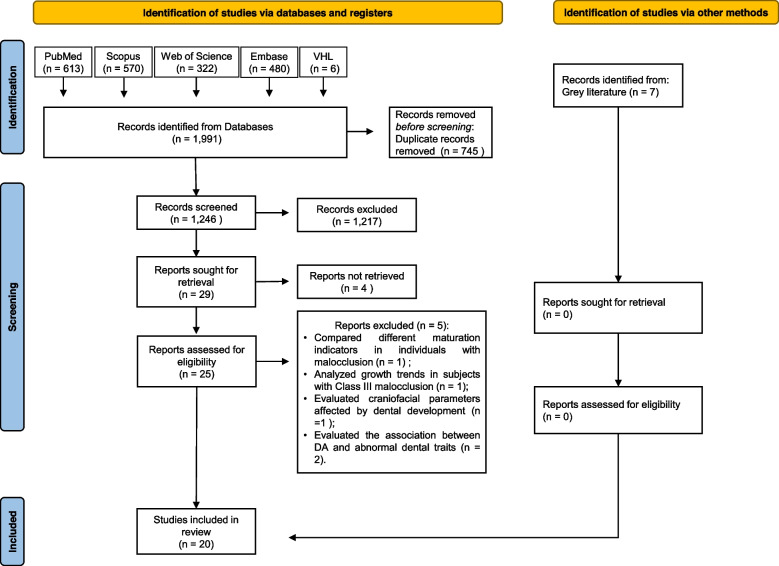
PRISMA 2020 flow diagram

A total of 1,991 studies were identified in the initial search. After removing duplicates, 1,246 studies remained. Sixty studies were selected by title and abstract. Of them, twenty-five were eligible for full-text evaluation. Then, five studies were excluded because they did not answered our focused question: one aimed to compare different maturation indicators in individuals with malocclusion [25]; one analyzed growth trends in subjects with Class III malocclusion [26]; one evaluated craniofacial parameters affected by dental development [27]; and two studies evaluated the association between DA and abnormal dental traits [28, 29]. Thus, twenty studies were included in this systematic review (Fig. 1).

### Characteristics of included studies

All 20 included studies were cross-sectional. Three studies were conducted in Brazil [14, 16, 30], four in Turkey [13, 31, 32], three in India [33–35], two in Pakistan [36, 37], two in South Korea [5, 38], one in Bosnia and Herzegovina [6], one in Japan [40], one in Netherlands [41], one in Poland [15], one in Ukraine [42], one in Israel and Turkey [43]. Patients’ ages ranged from 7 to 19 years old. The studies included sample sizes of 40 [16] to 776 [6] participants, respectively (Table 1).

**Table 1:** General characteristics of included studies and assessment of the risk of bias according to the Newcastle−Ottawa Scale (NOS)

**Study ID**	**Country**	**Age of paticipants [mean±SD]**	**Total number of participants**	**% of male paticipants**	**Setting of recruitment**	**Groups (n)**	**NOS**			
							**S**	**C**	**O**	**Total**
Akturk et al, 2021 [39]	Turkey	NR	80	32.5%	University	Dental Class I (42) Dental Classe II (38)	*	*	**	4
Amaral et al, 2019 [14]	Brazil	8 to 12 [NR]	200	45.0%	Schools	Dental Class I (100) Dental Class II (100)	**	*	**	5
Brin et al, 2006 [43]	Israel and Turkey	7 to 17 [11.3]	221	41.6%	Orthodontic clinic	Skeletal Class I (41) Maxillary class II (68) Mandibular class II (112)	*	*	**	4
Celikoglu et al, 2011 [13]	Turkey	9 to 15 [12.51]	525	48.8%	University	Skeletal Class I (162) Skeletal Class II (186) Skeletal Class III (177)	****	*	*	6
Durka-Zając et al, 2017 [15]	Poland	9 to 12 [9.83]	150	50.0%	Orthodontic clinic	Skeletal Class I (50) Skeletal Class II (50) Skeletal Class III (50)	*	*	*	3
Esenlik, Atak and Altun, 2014 [31]	Turkey	7 to 15 [NR]	321	48.6%	University	Skeletal Class I (107) Skeletal Class II (152) Skeletal Class III (62)	***	**	**	7
Goncharuk-Khomyn et al, 2020 [42]	Ukraine	15 to 17 [NR]	61	NR	University	Class I (23) Class II (19) Class III (19)	**	*	*	4
Gottimukkala et al., 2012 [33]	India	9 to 12 [NR]	100	50.0%	Private schools	Vertical growth pattern (50) Horizontal growth pattern (50)	*	*	*	3
Goyal et al, 2011 [34]	India	8 to 10 [NR]	150	50.0%	University	Normal growth pattern (50) Vertical growth pattern (50) Horizontal growth pattern (50)	**	*	*	4
Haruki, Kanomi and Shimono, 1997 [40]	Japan	NR	53	43.4%	Orthodontic clinic	Dental Class II (27) Dental Class III (26)	**		**	4
Jamroz et al, 2006 [41]	Netherlands	9 to 12 [11.3±1]	176	NR	University	Vertical growth pattern (107) Horizontal growth pattern (69)	***	*	**	6
Janson et al., 1998 [16]	Brazil	7 to 10 [9]	40	50.0%	University	Vertical growth pattern (20) Horizontal growth pattern (20)	*	*	**	4
Jeong and Yang, 1996 [38]	South Korea	8 to 13 [10]	333	50.8%	University	Dental Class I (182) Dental Class III (151)	*	**	*	4
Jo et al., 2021 [5]	South Korea	8 to 14 [NR]	184	52.2%	University	Normal growth pattern (93) Vertical growth pattern (49) Horizontal growth pattern (42)	***	*	**	6
Kamble et al., 2014 [35]	India	8 to 14 [NR]	60	NR	NR	Normal growth pattern (20) Vertical growth pattern (20) Horizontal growth pattern (20)		*		1
Lauc et al., 2017 [6]	Bosnia and Herzegovina	7 to 15 [NR]	776	47.4%	University	Skeletal Class I (284) Skeletal Class II (218) Skeletal Class III (274)	***	*	**	6
Mahmood and Fida, 2018 [36]	Pakistan	9 to 16 [NR]	270	50.0%	University	Dental Class I (100) Dental Class II (100) Dental Class III (70)	***	**	*	6
Neves et al, 2005 [30]	Brazil	8 to 8.92 [NR]	60	50.0%	University	Vertical growth pattern (30) Horizontal growth pattern (30)	***	*	**	6
Sukhia and Fida, 2010 [37]	Pakistan	7 to 17 [NR]	264	42.0%	University	Normal growth pattern (88) Vertical growth pattern (88) Horizontal growth pattern (88) Skeletal Class I (132) Skeletal Class II (132)	****	*	**	7
Uysal, Yagci and Ramoglu, 2005 [32]	Turkey	8 to 13 [10.90±1.62]	101	52.5%	University	Dental Class I (50) Unilateral posterior crossbite (51)	*	**	**	5

*NR* Not reported, *S* Selection, *C* Comparability, *O* Outcome

Most of the included studies recruited the patients from universities [5, 6, 13, 16, 30–32, 34, 36–39, 41, 42]. Three studies recruited patients from orthodontic clinics [15, 40, 43]; two studies recruited patients from schools [14, 33]; and one did not report the setting of participant recruitment [35] (Table 1).

Twelve studies evaluated the association between DA and sagittal discrepancies [6, 13–15, 31, 36–40, 42, 43]; eight studies evaluated vertical discrepancies [5, 16, 30, 33–35, 37, 41]; and only one study analyzed a transversal discrepancy (unilateral posterior cross-bite) [32]. Regarding the sagittal discrepancies, five studies used the Angle’s classification for malocclusion [14, 36, 38–40]; five used the ANB in cephalometric analysis to classify the skeletal malocclusion [6, 13, 31, 37, 43]; one considered the ANPg angle [44] in cephalometric analysis [15]; and one did not report the criteria adopted to classify the sagittal malocclusion [42].

Vertical discrepancies were mainly evaluated considering the ratio of Lower Anterior Face Height and Total Anterior Face Height (LAFH : TAFH) [16, 33, 37, 41]; three studies considered other cephalometric measurements (SNGoGn angle, Frankfort mandibular angle, and Jaraback ratio) [5, 30, 34]; and one study did not report which measurements were used [35]. The study that evaluated unilateral posterior crossbite included patients with at least a crossbite of two lower posterior teeth in one side in combination with a mandibular dental midline deviation of at least 1 mm [29] (Table 1).

All included studies used panoramic radiographs to evaluate DA. The majority of studies used the system proposed by Demirjian, Goldstein and Tanner (1973) [45] to evaluate DA [5, 13–16, 30–39, 41, 42], but among these studies, Akturk et al. (2021) evaluated only third molars, and Jeong and Yang evaluated only the lower left canine. Two studies used the Nolla method (1960) [46] and evaluated only the stages of development of second molars [40, 43]. One study [6] used both Willems [47] and Cameriere [48] methods. The general characteristics of included studies are presented in Table 1.

### Synthesis of results

#### Sagittal discrepancies

The association between DA and sagittal discrepancies could be only qualitatively analyzed. Two of the included studies found that patients presenting Class II malocclusion showed a lower DA in comparison to the other groups [14, 15]. One study did not find a difference in DA among the sagittal malocclusions evaluated [42]. However, it is important to point out that Amaral et al. [14] evaluated dental malocclusions, while Durka-Zając et al. [15] and Goncharuk-Khomyn et al. [42] evaluated skeletal malocclusions (Table 2).

**Table 2:** Data about chronological age (CA) and dental age (DA) among the studies that evaluated sagittal discrepancies

**Study ID**	**Criteria for DA evaluation**	**Malocclusion evaluated (criteria)**	**N**	**Mean CA**	**SD**	**Mean DA**	**SD**	**DA-CA**	**SD**	**Description of Results**
Amaral et al., 2019 [14]	Demirjian	Class I (Angle)	77	11.50	0.86	10.87	0.86	NR	NR	• Patients with Angle Class II, division two malocclusion presented lower DA than patients with Angle Class I malocclusion.
		Angle Class II, 2 (Angle)	95	11.45	1.55	10.19	1.22	NR	NR	
Durka-Zając et al, 2017 [15]	Demirjian	Class I (ANPg = -0.5-4º)	50	NR	NR	9.55	1.71	NR	NR	• Patients with skeletal Class II malocclusion presented the lower DA among the groups. • Patients with skeletal Class III presented a significantly higher DA than patients with skeletal Class II.
		Class II (ANPg >4º)	50	NR	NR	9.51	1.31	NR	NR	
		Class III (ANPg <-0.5)	50	NR	NR	10.44	1.6	NR	NR	
Goncharuk-Khomyn et al., 2020 [42]	Demirjian	Class I (NR)	23	NR	NR	NR	NR	0.49	0.35	• No significant difference in DA was found among the groups.
		Class II (NR)	19	NR	NR	NR	NR	0.37	0.69	
		Class III (NR)	19	NR	NR	NR	NR	0.64	0.47	
Sukhia and Fida, 2010 [37]	Demirjian	Class I (ANB=0-4º)	132	NR	NR	13.03	2.29	NR	NR	• No significant difference in DA was found among the groups.
		Class II (ANB>4º)	132	NR	NR	12.86	2.16	NR	NR	

*SD* Standard deviation, *NR* Not reported., *CA* Chronological age, *DA* Dental age

Akturk et al. [39] evaluated the DA of third molars in patients with unilateral Class II malocclusions. They did not find a difference in DA between jaw sides and not in comparison to a symmetric Class I control group. Brin et al. [43] compared Class I and Class II skeletal malocclusions considering the development of second molars. They did not find an association between DA and the type of malocclusion, either (Table 2).

Some studies evaluated DA according to the type of sagittal discrepancy and the patient’s sex. About skeletal malocclusions, Celikoglu et al. [13] reported that in both sexes, Class III presented the most advanced DA. Esenlik et al. [31] and Lauc et al. [6] reported that male Class III patients presented the most advanced DA in comparison to the other skeletal malocclusion groups. The results for females are controversial; in the Esenlik et al. study [31], the Class II group presented the most advanced DA; in Lauc et al. [6] no difference was observed between the malocclusions using both Willems’ and Cameriere’s methods. Mahmood et al. [36] considered the dental malocclusions classified by Angle and observed that Class I and Class III individuals in the male sample presented with a significantly higher DA than Class II. In the female sample, no difference was found by the authors (Table 3).

**Table 3:** Data about chronological age (CA) and dental age (DA) among the studies that evaluated sagittal discrepancies in relation to sex

**Study ID**	**Criteria for DA evaluation**	**Malocclusion evaluated (criteria)**	**Sex**	**N**	**Mean CA**	**SD**	**Mean DA**	**SD**	**DA-CA**	**SD**	**Description of results**
Celikoglu et al., 2011 [13]	Demirjian	Class I (ANB=0-4º)	Male	77	12.59	NR	13.17	NR	0.58	NR	• In both sexes, patients with Class III skeletal malocclusion presented the most advanced DA. • DA was overestimated in this sample in relation to the CA.
			Female	85	13.01	NR	13.64	NR	0.63	NR	
		Class II (ANB > 5º)	Male	95	12.75	NR	13.85	NR	1.1	NR	
			Female	91	12.39	NR	13.47	NR	1.08	NR	
		Class III (ANB <0)	Male	84	12.45	NR	13.6	NR	1.15	NR	
			Female	93	11.39	NR	12.77	NR	1.38	NR	
Esenlik, Atak and Altun, 2014 [31]	Demirjian	Class I (ANB=NR)	Male	49	11.71	1.65	12.05	1.71	0.34	0.75	• In the male group, Class III patients presented the most advanced DA in relation to the other skeletal malocclusion. • In the female group, Class II patients presented the most advanced DA in relation to the other skeletal malocclusion. • DA was overestimated in this sample in relation to the CA.
			Female	58	11.57	1.85	12.18	1.94	0.61	1.28	
		Class II Class I (ANB=NR)	Male	75	12.29	1.41	12.49	1.31	0.2	0.79	
			Female	77	11.61	1.42	12.66	1.65	1.05	0.85	
		Class III Class I (ANB=NR)	Male	32	10.98	1.44	11.35	1.6	0.37	1	
			Female	30	10.44	1.81	11.24	1.91	0.8	1.03	
Lauc et al., 2017 [6]	Willems	Class I (ANB=0-4º)	Male	136	11.71	1.94	12.11	2.54	0.4	1.13	• DA was overestimated in this sample in relation to the CA. • In the male group, Class III patients presented the most advanced DA in relation to the other skeletal malocclusion. • In the female group, no significant difference in DA was observed among the skeletal malocclusions.
			Female	148	12	2.01	12.52	2.5	0.53	1.12	
		Class II (ANB > 5º)	Male	100	11.67	2	12.11	2.54	0.44	1.03	
			Female	118	11.96	1.86	12.38	2.53	0.43	1.17	
		Class III (ANB ≤0)	Male	132	11.96	2.17	12.79	2.65	0.83	0.97	
			Female	142	12.19	1.96	12.68	2.57	0.49	1.07	
Lauc et al., 2017 [38]	Cameriere	Class I (ANB=0-4º)	Male	136	11.71	1.94	11.44	2.04	-0.26	0.72	• DA was underestimated in this sample in relation to the CA. • In the male Class III group, the underestimation of the DA was less prominent as compared to the other malocclusion groups. • In the female group, no significant difference in DA was observed among the skeletal malocclusions.
			Female	148	12	2.01	11.86	1.7	-0.14	0.91	
		Class II (ANB > 5º)	Male	100	11.67	2	11.44	1.9	-0.23	0.76	
			Female	118	11.96	1.86	11.75	1.72	-0.20	0.73	
		Class III (ANB ≤0)	Male	132	11.96	2.17	11.93	1.99	-0.02	0.73	
			Female	142	12.19	1.96	11.94	1.74	-0.24	0.73	
Mahmood and Fida, 2018 [36]	Demirjian	Class I (Angle)	Male	NR	NR	NR	13.53	2.3	NR	NR	• In the male group, Class I and Class III patients presented a significantly higher DA than Class II patients. • In the female group, no difference was found.
			Female	NR	NR	NR	13.41	2.22	NR	NR	
		Class II (Angle)	Male	NR	NR	NR	12.57	2.03	NR	NR	
			Female	NR	NR	NR	13.34	2.15	NR	NR	
		Class III (Angle)	Male	NR	NR	NR	13.64	2.07	NR	NR	
			Female	NR	NR	NR	13.59	2	NR	NR	

*SD* Standard deviation, *NR* Not reported., *CA* Chronological age, *DA* Dental age

Celikoglu et al. [13] and Esenlik et al. [31] found an overestimated DA when compared CA considering both males and females and the three types of skeletal malocclusions. Unlike these studies, which used the Demirjian criteria to evaluate DA, Lauc et al. [6] used the methods of Willems and Cemeriere and observed contrasting results between the methods. When using the Willems criteria, the authors also observed an overestimated DA comparing to the CA in both sexes and in all types of skeletal malocclusions. However, with Cameriere’s method, opposite results were found (Table 3).

Haruki, Kanomi, and Shimono [40] evaluated the development of second molars in Class II and Class III dental malocclusions. The authors found no difference regarding DA among the malocclusions both sexes’ groups. Jeong and Yang [38] compared Class I and Class II dental malocclusions considering only the left lower canine and observed no difference in the development stage of this tooth between the groups (Table 3).

#### Vertical discrepancies

Most of the studies that evaluated the association between DA and vertical discrepancies observed a greater DA in the vertical groups [5, 16, 30, 33–35]. Only Jamroz et al. [41] and Sukhia and Fida [37] did not find differences in DA among different vertical growth patterns. These studies, however, adopted different measures and cut-off points to classify the vertical discrepancies (Tables 4 and 5).

**Table 4:** Data about chronological age (CA) and dental age (DA) among the studies that evaluated vertical discrepancies

**Study ID** [30]	**Criteria for DA evaluation**	**Malocclusion evaluated (criteria)**	**N**	**Mean CA**	**SD**	**Mean DA**	**SD**	**DA-CA**	**SD**	**Description of results**
Jamroz at al., 2006 [41]	Demirjian	Vertical (LAFH/TAFH ≥58º%)	107	11.00	1.00	11.60	1.20	0.60	1.0	• No difference was observed between the groups.
		Horizontal (LAFH/TAFH ≤56º)	69	11.40	0.80	12.10	1.20	0.70	1.0	
Janson et al., 1998 [16]	Demirjian	Vertical (LAFH/TAFH= NR)	20	9.20	1.05	10.04	1.69	NR	NR	• The vertical group presented a significantly advanced DA in comparison to the horizontal group.
		Horizontal (LAFH/TAFH= NR)	20	9.25	1.02	9.50	1.38	NR	NR	
Jo et al., 2021 [5]	Demirjian	Normal (SN-GoMe= 33-40º)	93	11.20	1.70	12.70	2.22	NR	NR	• DA was highest in the vertical growth group. • The vertical growth group showed significantly greater DA than the normal growth group. • The difference in DA was not statistically significant when assessed between the horizontal growth and the normal growth groups.
		Vertical (SN-GoMe ≥41º)	49	11.60	1.70	13.67	1.94	NR	NR	
		Horizontal (SN-GoMe <33º)	42	11.70	1.70	12.11	1.83	NR	NR	
Kamble et al., 2014 [35]	Demirjian	Normal (NR)	20	10.90	0.47	11.49	0.74	NR	NR	• The vertical group presented a significantly advanced DA in comparison to the horizontal group.
		Vertical (NR)	20	10.14	0.98	10.67	1.02	NR	NR	
		Horizontal (NR)	20	10.24	1.45	9.66	1.46	NR	NR	
Neves et al., 2005 [30]	Demirjian	Vertical^a^	30	8.47	NR	8.94	0.75	NR	NR	• The vertical group presented a significantly advanced DA in comparison to the horizontal group.
		Horizontal^a^	30	8.42	NR	8.17	0.48	NR	NR	
Sukhia and Fida, 2010 [37]	Demirjian	Normal (LAFH/TAFH 56-58%)	88	NR	NR	12.94	2.17	NR	NR	• No difference was observed among the groups.
		Vertical (LAFH/TAFH ≥59%)	88	NR	NR	12.68	2.33	NR	NR	
		Horizontal (LAFH/TAFH <55%)	88	NR	NR	13.22	2.17	NR	NR	

*SD* Standard deviation, *NR* Not reported., *CA* Chronological age, *DA* Dental age, *LAFH/TAFH* Lower anterior facial height/ Total anterior facial height

^a^Neves used the following parameter to classify the type of vertical malocclusion: SNGoGn, NS-Gn, Frankfort-mandibular angle, and LAFH. These measures were standardized to create a level playing field where all 4 values could contribute equally to classify the growth pattern. The standardized variables were summed for each subject

**Table 5:** Data about chronological age (CA) and dental age (DA) among the studies that evaluated vertical discrepancies related to sex

**Study ID**	**Criteria for DA evaluation**	**Malocclusion evaluated (criteria)**	**Sex**	**N**	**Mean CA**	**SD**	**Mean DA**	**SD**	**DA-CA**	**SD**	**Description of results**
Gottimukkala et al., 2012 [33]	Demirjian	Vertical (LAFH/TAFH ≥58%)	Male	25	10.72	0.98	11.74	1.13	NR	NR	• In the vertical group, both genders presented advanced DA in relation to the horizontal group.
			Female	25	10.20	1.00	11.13	1.19	NR	NR	
		Horizontal (LAFH/TAFH ≤56%)	Male	25	11.04	0.89	10.30	1.34	NR	NR	
			Female	25	10.72	1.02	9.78	1.12	NR	NR	
Goyal et al., 2011 [34]	Demirjian	Normal (SN-GoGn 32+2º; LAFH 60-62 mm; Jarabak ratio 62-65%)	Male	25	9.58	NR	9.57	NR	NR	NR	• In the vertical group, both genders presented advanced DA in relation to the horizontal group. • There no significant differences were found between vertical growing patients and horizontal growing patients were compared to patients with a normal growth pattern. • No difference was found in DA between males and females when compared inside the same growth pattern.
			Female	25	9.33	NR	9.36	NR	NR	NR	
		Vertical (SN-GoGn >34º; LAFH >62 mm; Jarabak ratio <62%)	Male	25	9.42	NR	9.94	NR	NR	NR	
			Female	25	9.06	NR	9.68	NR	NR	NR	
		Horizontal (SN-GoGn >30º; LAFH <60 mm; Jarabak ratio >65%)	Male	24	9.70	NR	9.22	NR	NR	NR	
			Female	25	9.43	NR	9.05	NR	NR	NR	
Janson et al., 1998 [16]	Demirjian	Vertical (LAFH/TAFH= NR)	Male	10	9.43	1.13	10.48	1.79	NR	NR	• DA had a tendency to be more advanced in both genders in the vertical group as compared with the horizontal group.
			Female	10	8.98	0.96	9.60	1.55	NR	NR	
		Horizontal (LAFH/TAFH= NR)	Male	10	9.51	1.11	9.96	1.40	NR	NR	
			Female	10	8.98	0.89	9.04	1.27	NR	NR	
Jo et al., 2021 [5]	Demirjian	Normal (SN-GoMe 33-40º)	Male	44	11.30	1.50	12.91	2.16	NR	NR	• No difference was found among growth patterns and between genders.
			Female	49	11.00	1.90	12.52	2.29	NR	NR	
		Vertical (SN-GoMe >41º)	Male	25	11.80	1.60	13.54	1.98	NR	NR	
			Female	24	11.30	1.70	13.80	1.94	NR	NR	
		Horizontal (SN-GoMe <33º)	Male	27	12.00	1.30	12.23	1.74	NR	NR	
			Female	15	11.10	2.10	11.91	2.03	NR	NR	

*SD* Standard deviation, *NR* Not reported., *CA* Chronological age, *DA* Dental age, *LAFH/TAFH* Lower anterior facial height/ Total anterior facial height

#### Transversal discrepancy

Only one study that met the eligibility criteria of this systematic review investigated a transversal discrepancy [32]. The authors reported that DA tended to be delayed in the posterior-cross bite group as compared to the non-cross bite group (Table 6).

**Table 6:** Data about chronological age (CA) and dental age (DA) in the study that evaluated a transversal discrepancy

**Study ID**	**Criteria for DA evaluation**	**Malocclusion evaluated (criteria)**	**Sex**	**N**	**Mean CA**	**SD**	**Mean DA**	**SD**	**DA-CA**	**SD**	**Description of results**
Uysal, Yagci and Ramoglu, 2005 [32]	Demirjian	Unilateral posterior crossbite (Unilateral posterior crossbite involving at least two posterior teeth combined with a mandibular dental midline deviation of at least 1 mm to the crossbite side)	Male	23	10.88	1.76	11.38	1.55	NR	NR	• Patients with a posterior crossbite had a tendency for a delayed DA compared to the patients without posterior crossbite. • No difference was observed between the sexes.
			Female	28	10.87	2.22	11.44	2.78	NR	NR	
			Total	51	10.87	2.01	11.42	2.65	NR	NR	
		Non-crossbite (Angle Class I, normal overjet and overbite, and coincidence of both dental midlines)	Male	25	10.99	1.02	14.06	4	NR	NR	
			Female	25	10.88	1.27	13.89	1.67	NR	NR	
			Total	50	10.93	1.14	13.97	3.03	NR	NR	

*SD* Standard deviation, *NR* Not reported., *CA* Chronological age, *DA* Dental age

### Quality assessment

According to NOS, three studies presented low quality [15, 33, 35], fifteen presented moderate quality [5, 6, 13, 14, 16, 30, 32, 34, 36, 38–43], and two presented high quality [31, 37]. Only four studies received two stars in the comparability dimension (Table 1).

### Certainty of evidence

The certainty of evidence was very low for all outcomes evaluated (Tables 7 and 8). Regarding the association between DA and the types of malocclusions, not considering the sex, the risk of bias domain was classified as serious for sagittal, vertical, and transversal discrepancies because most of the studies included presented a moderate risk of bias. Once the studies that evaluated sagittal discrepancies showed contrasting findings, the inconsistency domain was rated as serious.

**Table 7:** Assessment of certainty of evidence of the evaluation of the association between malocclusion and dental age (GRADE)

**Certainty assessment**						
**Participants** **(studies)** **Follow-up**	**Risk of bias**	**Inconsistency**	**Indirectness**	**Imprecision**	**Publication bias**	**Overall certainty of evidence**
**Sagittal discrepancies**						
647 (4 observational studies)	serious^a^	serious^b^	not serious	not serious	none	⨁◯◯◯ Very low
**Vertical discrepancies**						
784 (6 observational studies)	serious^a^	not serious	not serious	not serious	none	⨁◯◯◯ Very low
**Transversal discrepancy**						
101 (1 observational study)	serious^c^	not serious	not serious	serious^d^	none	⨁◯◯◯ Very low

^a^Most of the studies included presented a moderate quality

^b^The studies included presented different directions of effect

^c^The study presented a moderate quality

^d^The optimal information size (≥400) was not attended

**Table 8:** Assessment of certainty of evidence of the evaluation of the association between malocclusion and dental age considering the sex (GRADE)

**Certainty assessment**						
**Participants** **(studies)** **Follow-up**	**Risk of bias**	**Inconsistency**	**Indirectness**	**Imprecision**	**Publication bias**	**Overall certainty of evidence**
**Sagittal discrepancies in males**						
1148* (4 observational studies)	serious^a^	serious^b^	not serious	not serious	none	⨁◯◯◯ Very low
**Sagittal discrepancies in females**						
1250* (4 observational studies)	serious^a^	serious^b^	not serious	not serious	none	⨁◯◯◯ Very low
**Vertical discrepancies in males**						
240 (4 observational studies)	very serious^c^	not serious	not serious	serious^d^	none	⨁◯◯◯ Very low
**Vertical discrepancies in females**						
233 (4 observational studies)	very serious^c^	not serious	not serious	serious^d^	none	⨁◯◯◯ Very low

^a^Most of the studies included presented a moderate risk of bias

^b^The studies included presented different directions of effect

^c^The studies included presented a moderate or high risk of bias

^d^The optimal information size (≥400) was not attended

^*^One study did not present the number of participants per group

About the evaluations that considered the patient's sex, the risk of bias domain was classified as serious and very serious for sagittal and vertical discrepancies, respectively. Most studies that evaluated the association between DA and sagittal malocclusions presented a moderate risk of bias. The studies that assessed the association between DA and vertical discrepancies demonstrated a moderate or high risk of bias. The indirectness domain was rated as serious only for the evaluation of sagittal discrepancies in females because the studies included had controversial results. The optimal information size (n > 400) was not attempted in the evaluation of vertical discrepancies in both males and females; thus, the imprecision domain was classified as serious.

## Discussion

This systematic review aimed to investigate if DA varies in different types of malocclusions. Our results from the primary studies showed that DA may be associated with some types of malocclusions. The literature suggests that the type of sagittal [6, 13, 15, 31, 36], vertical [5, 16, 30, 33–35] and also in the transversal [32] malocclusions are associated with DA. Although the literature suggests the association between both conditions, the nature of this association and that factors involved in the connection between DA and craniofacial patterns/skeletal malocclusions remains unclear. Several genes are expressed during the craniofacial development and dental development. Some of these genes that have a biologically pleiotropic effect on both dental arches and dental development could explain the connection between these two traits. It is also possible that once the permanent tooth germ acts as a functional matrix, dental development would contribute to the sagittal and vertical growth of the maxilla and mandible [27].

The primary studies included in this systematic review reflects the orthodontic literature, in which most of the studies explored the association between sagittal or vertical malocclusion and DA. Only two of the included studies [14, 15] found a significant association between sagittal discrepancies and DA. They observed that patients with Class II presents a lower DA comparing to the others sagittal discrepancies. The sagittal disorders can be classified with regards to dental malocclusions and skeletal morphology. Some studies [6, 13, 15, 31, 37, 43] investigated the skeletal sagittal malocclusions that are characterized by a sagittal discrepancy between the maxilla and mandible [49]. These discrepancies are commonly investigated in cephalometric radiographs. The dental sagittal malocclusions classification is essentially based on Angle’s classification that is based on the anteroposterior relationship of the maxillary and mandibular first permanent molars [50]. Although the evaluation of the malocclusion based on the dental relationship has several limitations, this method was used by 5 included studies [14, 36, 38–40]. One study [42] did not report if dental or skeletal was used to investigate the outcome. It is important to emphasize that the results of primary studies are not consistent, regarding the sagittal discrepancies.

It is known that the sex influences teeth development [51] and dental arches [52] Therefore, some of primary included studies evaluated the data stratified according to the sex [6, 13, 31, 36]. The studies that evaluated the association between DA and sagittal malocclusions stratified by the sex observed that boys with skeletal Class III presented a more advanced DA than boys with other types of skeletal sagittal discrepancies [6, 13, 31]. On the other hand, for girls, the results were not conclusive. Among the studies that evaluated the association between DA and malocclusions [14, 15, 37, 42] regardless the sex.

Vertical malocclusions were also investigated in some of the included studies. Unlike the studies exploring the sagittal discrepancies, the studies about the association between DA and vertical discrepancies presented consistent results. Individuals with vertical growth patterns tended to have advanced DA than those with horizontal growth patterns [5, 16, 30, 33–35]. When evaluating the association between DA and vertical discrepancies considering the sex, similar results were observed – both males and females with vertical growth pattern presented advanced DA [5, 16, 33, 34]. The idea that patients with different vertical facial types present with a different timing of their adolescent growth spurt is well established in the literature. Those with a vertical growth pattern tend to begin their growth spurt, especially in the facial structures, earlier than those with a horizontal growth pattern [53]. This advanced development may explain the association between the vertical pattern and advanced DA.

One important limitation to be highlighted is that although most of the studies adopted the LAFH:TAFH ratio [16, 33, 37, 41] to evaluate the vertical discrepancies, the cut off values diverged among the included studies. Thus, one patient could be classified as presenting a normal growth pattern in one study and presenting a vertical growth pattern in another study, which may impact in the interpretation of the results.

Malocclusions that involve the transverse dimension are very common in the orthodontic office and include both malocclusions in the posterior and anterior region of the dentition [54]. Only one included study investigated a transversal discrepancy, the unilateral posterior crossbite [32]. The authors reported that DA tended to be more delayed in the posterior-cross bite group than in the non-cross bite group and suggested that this association could be explained with the individual genetic background [32], but it is also possible that some local factors could be involved in this delay. A previous study reported that in some patients the posterior crossbite has a genetic background and is associated with a narrow maxilla [54]. However, a study with twins demonstrated non-significant genetic variance for posterior crossbite 1990 [55]. It is well known that twin studies are a special type of epidemiological studies designed to measure the contribution of genetics and environmental factors to a given characteristic [56]. Although Uysal et al. (2005) [32] reported that patients with a posterior crossbite had a tendency for a delayed DA compared to the patients without posterior crossbite, their result should be interpreted with caution. In most cases, transverse malocclusions do not exist as a separate entity but are commonly associated with additional alterations in both the sagittal and vertical dimension [54]. The frequency of posterior cross bite is greater in patients presenting with a horizontal growth pattern than in patients with vertical skeletal growth patterns [57]. As mentioned in this review, the horizontal growth pattern is associated with delayed DA. Thus, a possible association between the horizontal growth patterns with posterior cross-bite could explain the delayed DA between patients with unilateral posterior crossbite. Therefore, it is important to highlight that more studies are necessary to confirm their findings. It is also important to highlight that in future studies the discrepancies in the different planes should be considered together.

In literature, various methods were described for DA assessment, such as Demirjian [45], Nolla [46], Willems [47] and Cameriere [48] methods. Most of the studies included in this systematic review used the Demirjian criteria. This method has been considered as the most widely accepted method for DA estimation and has been widely used in different populations [8]. A systematic review evaluated accuracy of the Demirjian’s method and observed that it overestimated the age by about half a year for both genders. Even if there are some geographical/ethnic differences, they are rather small, making the method useful for different populations [58].

Demirjian’s method was formulated on a sample of French-Canadian children. It assesses eight specific stages of dental formation of the seven left mandibular permanent teeth. Biologic weights are assigned to each tooth stage and added together to give a dental maturity score [33], and then separate tables of dental maturity for males and females are used to convert the maturity scores to dental age. Two studies used the Nolla method (1960) [46], and one study [6] used both Willems [47] and Cameriere [48] methods. Willems and colleagues [47] modified the Demirjian method by creating new tables from which a maturity score could be directly expressed in years. The step of converting the maturity score to a DA was omitted, making the new method simpler to use while retaining the advantages of Demirjian’s method [59]. Cameriere’s method assesses age based on the measurement of the open apices in teeth [48]. Similar to Demirjian’s method, the Nolla’s method [46] assesses the degree of dental development of the mandibular and maxillary teeth on the left side (excluding the third molars) by classifying them into ten degrees of dental development. A score is assigned to each tooth, which is converted to an average score according to sex. All the values are added, and the result corresponds to the dental age [60]. A previous study concluded that while Demirjian’s and Willem’s methods overestimated the children’s age, Cameriere’s method underestimated [61].

It is important to raise the limitation of this study, in which only two studies presented high quality according to NOS [31, 37]. In general, the included studies presented an unrepresentative sample and the absence of sample size calculation. Besides that, some of them did not describe appropriately the statistical data, such as the mean difference between DA and CA of the total sample and the standard deviation of DA. Consequently, it was not possible to perform a meta-analysis.

The certainty of evidence was very low for all evaluations performed in this study, which means that the true effect is likely to be substantially different from the estimate of effect [22]. In the GRADE approach [22], the evidence from observational studies is initially classified as low due to the inherent limitations of this type of study design. Besides that, the rating of the domains of this tool may affect the overall certainty of evidence. In all the evaluations of the association between the types of malocclusions and DA, the risk of bias was rated as “serious” or “very serious” because most of the primary studies included here presented a moderate or low methodological quality according to the NOS. The inconsistency was rated as “serious” for sagittal discrepancies due to the contrasting findings among the studies, which may be related to the characteristics of the samples included, and the different methods used for DA assessment among the studies. The population, exposure, and outcome evaluated in the primary studies provided direct evidence for the research question, so the indirectness domain was rated as “not serious” in all the evaluations performed. The imprecision was rated as “serious” for the evaluations of the traversal discrepancy despite the sex and for the vertical discrepancies considering the sex because the OIS was not attempted by the primary studies. The publication bias was rated as “none” for all evaluations, since the primary studies included here presented both positive and negative results and were published, not only reported in registers.

Deciding the timing of clinical interventions in functional and preventive orthodontic treatment approaches is critical for achieving successful outcomes in the treatment of different types of malocclusions [15, 31]. The ideal period for beginning dental treatments, such as orthodontic or orthopedic treatments may change according to the patient’s malocclusion. Based on the results observed in the present study the orthodontist and pediatric dentists should keep in mind that time of clinical treatment should change according to the patients’ characteristics and malocclusion. Males with skeletal class III malocclusion and patients with a predominantly vertical growth pattern could present with a more advanced DA in comparison to their CA than patients with other types of malocclusions. Our results suggest that the evaluation of the DA can be a useful initial diagnostic tool when assessing jaw development and treatment planning.

## Conclusions

Males with skeletal class III malocclusion and patients with a predominantly vertical growth pattern could present with a more advanced DA in comparison to their CA than patients with other types of malocclusions. Future well designed studies should be performed to investigate the association between DA and different malocclusions in more detail.

### Supplementary Information

**Supplementary Material 1.**

## Data Availability

No datasets were generated or analysed during the current study.

## Abbreviations

CA: Chronological age
DA: Dental age
GRADE: Grading Recommendations Assessment, Development and Evaluation
IADR: International Association of Dental Research
LAFH/TAFH: Lower anterior facial height/ Total anterior facial height
NOS: Newcastle−Ottawa Scale
PRISMA: Preferred Reporting Items for Systematic Review and Meta-Analysis
PROSPERO: International Prospective Register of Systematic Reviews
SD: Standard Deviation
