# Supplementary material for: Association between dental age and malocclusions: a systematic review
Source: BMC Oral Health. 2024 Mar 25;24:383. doi: 10.1186/s12903-024-04143-7 (PMC10964512; doi:10.1186/s12903-024-04143-7)
Supplement: Supplementary file 1 — Supplementary Material 1. [file 12903_2024_4143_MOESM1_ESM.docx]

**Additional File 1** Search strategy.

| Database | Search Strategy |
| --- | --- |
| PubMed | ((((((((((((((((Dental Occlusion[MeSH Terms]) OR (Malocclusion[MeSH Terms])) OR (Dental Arch[MeSH Terms])) OR (Malocclusion, Angle Class I[MeSH Terms])) OR (Malocclusion, Angle Class II[MeSH Terms])) OR (Malocclusion, Angle Class III[MeSH Terms])) OR ("Dental Occlusion"[Title/Abstract])) OR (Malocclusion[Title/Abstract])) OR ("Dental Arch"[Title/Abstract])) OR ("Malocclusion, Angle Class I"[Title/Abstract])) OR ("Malocclusion, Angle Class II"[Title/Abstract])) OR ("Malocclusion, Angle Class III"[Title/Abstract])) OR ("Orthodontic treatment"[Title/Abstract])) OR (Orthodontics[Title/Abstract])) OR ("skeletal malocclusion"[Title/Abstract])) OR ("Occlusal alteration"[Title/Abstract])) AND ((((((((((((Calcification[MeSH Terms]) OR (Age Determination by Teeth[MeSH Terms])) OR (Odontogenesis[MeSH Terms])) OR (Calcification[Title/Abstract])) OR ("Age Determination by Teeth"[Title/Abstract])) OR (Odontogenesis[Title/Abstract])) OR ("Dental age"[Title/Abstract])) OR ("Dental maturation"[Title/Abstract])) OR ("Dental development"[Title/Abstract])) OR (Demirjian[Title/Abstract])) OR (Nolla[Title/Abstract])) OR (Willems[Title/Abstract])) |
| Scopus | ( ( TITLE-ABS-KEY ( "Dental Occlusion" ) OR TITLE-ABS-KEY ( malocclusion ) OR TITLE-ABS-KEY ( "Dental Arch" ) OR TITLE-ABS-KEY ( "Malocclusion, Angle Class I" ) OR TITLE-ABS-KEY ( "Malocclusion, Angle Class II" ) OR TITLE-ABS-KEY ( "Malocclusion, Angle Class III" ) OR TITLE-ABS-KEY ( "Orthodontic treatment" ) OR TITLE-ABS-KEY ( orthodontics ) OR TITLE-ABS-KEY ( "skeletal malocclusion" ) OR TITLE-ABS-KEY ( "Occlusal alteration" ) ) ) AND ( ( TITLE-ABS-KEY ( calcification ) OR TITLE-ABS-KEY ( "Age Determination by Teeth" ) OR TITLE-ABS-KEY ( odontogenesis ) OR TITLE-ABS-KEY ( "Dental age" ) OR TITLE-ABS-KEY ( "Dental development" ) OR TITLE-ABS-KEY ( demirjian ) OR TITLE-ABS-KEY ( nolla ) OR TITLE-ABS-KEY ( willems ) ) ) |
| Web of Science | #1 "Dental occlusion" (Tópico) or Malocclusion (Tópico) or "Dental arch" (Tópico) or "Malocclusion, Angle Class I" (Tópico) or "Malocclusion, Angle Class II" (Tópico) or "Malocclusion, Angle Class III" (Tópico) or "Orthodontic treatment" (Tópico) or Orthodontics (Tópico) or "skeletal malocclusion" (Tópico) or "Occlusal alteration" (Tópico)  #2 Calcification (Tópico) or "Age Determination by Teeth" (Tópico) or Odontogenesis (Tópico) or "Dental age" (Tópico) or "Dental maturation" (Tópico) or "Dental development" (Tópico) or Demirjian (Tópico) or Nolla (Tópico) or Willems (Tópico)  #1 AND #2 |
| Virtual Health Library | (mh:(Dental Occlusion)) OR ("Oclusão Dentária") OR ("Oclusión Dental") OR (mh:(Malocclusion )) OR ("Má Oclusão") OR (Maloclusión) OR (mh:(Dental Arch)) OR ("Arco dental") OR (mh:(Malocclusion, Angle Class I )) OR ("Má Oclusão Classe I de Angle") OR ("Maloclusión Clase I de Angle") OR (mh:(Malocclusion, Angle Class II)) OR ("Má Oclusão Classe II de Angle") OR ("Maloclusión Clase II de Angle") OR (mh:(Malocclusion, Angle Class III)) OR ("Má Oclusão Classe III de Angle") OR ("Maloclusión de Angle Clase III") OR ("Dental Occlusion") OR (Malocclusion) OR ("Dental Arch") OR ("Malocclusion, Angle Class I") OR ("Malocclusion, Angle Class II") OR ("Malocclusion, Angle Class III") OR ("Orthodontic treatment") OR ("Tratamento ortodôntico") OR ("tratamiento de ortodoncia") OR (Orthodontics ) OR (Ortodontia) OR (Ortodoncia) OR ("skeletal malocclusion") OR ("Maloclusão esquelética") OR ("Má oclusão esquelética") OR ("maloclusión esquelética") OR ("Occlusal alteration") OR ("Alteração occlusal") OR ("Alteración oclusal") AND (mh:(Calcification)) OR (mh:(Age Determination by Teeth)) OR (mh:(Odontogenesis )) OR (Calcification) OR ("Calcificação de Dente") OR ("Calcificación de Dientes") OR ("Age Determination by Teeth") OR ("Determinação da Idade pelos Dentes") OR ("Determinación de la Edad por los Dientes") OR (Odontogenesis ) OR (Odontogênese) OR ("Dental age") OR ("Idade dentaria") OR ("edad dental") OR ("Dental maturation") OR ("Maturação dentaria") OR ("maduración dental") OR ("Dental development") OR ("Desenvolvimento dentária") OR ("desarrollo dental") OR (Demirjian) OR (Nolla) OR (Willems) |
| Grey Literature | Malocclusion AND dental age  Malocclusion AND dental development  Dental age OR dental development |
